# Diagnosing *Capnocytophaga canimorsus* Infections

**DOI:** 10.3201/eid1202.050783

**Published:** 2006-02

**Authors:** J. Michael Janda, Margot H. Graves, David Lindquist, Will S. Probert

**Affiliations:** * California Department of Health Services, Richmond, California, USA

**Keywords:** Capnocytophaga, Zoonoses, Laboratory Techniques and Procedures, Dispatch

## Abstract

We reviewed clinical and epidemiologic features of 56 human *Capnocytophaga canimorsus* isolates submitted during a 32-year period to California's Microbial Diseases Laboratory for identification. An increasing number of isolates identified as *C. canimorsus* have been submitted since 1990. Many laboratories still have difficulty correctly identifying this species.

Dogs are the most common household pets in the United States. Estimates predict that 50% of all Americans will be bitten in their lifetime by an animal (*1*) and that ≈1 million dog bites will occur annually (*2*). *Capnocytophaga canimorsus* is the main human pathogen associated with dog bites; this organism causes septicemia, meningitis, endocarditis, and rare ocular infections (*3**,**4*). Persons at increased risk of developing *C. canimorsus* infections include patients who have undergone a splenectomy and those who abuse alcohol. We describe a series of 56 isolates submitted to California's Microbial Diseases Laboratory (MDL) in a 32-year period with laboratory and epidemiologic factors associated with these infections.

## The Study

MDL is California's reference laboratory for detecting and identifying bacterial, parasitic, and fungal infections of public health importance. Cultures submitted to MDL come from >500 clinical laboratories through a network of 39 county or city public health laboratories. Isolates for identification are forwarded to MDL by public health laboratories with standardized forms that include information on clinical condition or suspected disease, date of onset, a brief case history, antimicrobial therapy, origin of specimen, and laboratory results. In this manner, clinical information and patient demographics were obtained and analyzed for human cases of *C. canimorsus* infections identified from 1972 to 2004.

Confirmatory testing by our laboratory includes a combination of conventional and molecular techniques involving biochemical tests, fatty acid methyl ester analysis, and 16S rRNA gene sequencing. These procedures have been described in detail elsewhere (*5*). Morphologically, *Capnocytophaga* spp. appear as gram-negative medium-to-long rods with tapered or spindle-shaped ends. The major phenotypic characteristics of *C. canimorsus* include positive test results for oxidase, catalase, arginine dihydrolase, and *o*-nitrophenyl-β-d-galactopyranoside and negative reactions for urease, nitrates, and indole. Fermentation of glucose, lactose, and maltose is often observed but not of raffinose and inulin. Growth is often enhanced by the addition of rabbit serum and incubation in a carbon dioxide–enriched environment.

Sixty *C. canimorsus* isolates were forwarded to MDL for identification or confirmation during the 32-year period (1972–2004), 56 from humans and 4 from animals. The average number of clinical strains submitted per year was 1.75; the highest number was recorded in 1998 (n = 8). The average number of isolates from human cases forwarded to MDL increased from 1990 to 2004, when 2.2–2.5 strains were submitted each year, roughly a 4-fold increase over that observed in the 1970s (Figure).

**Figure:**
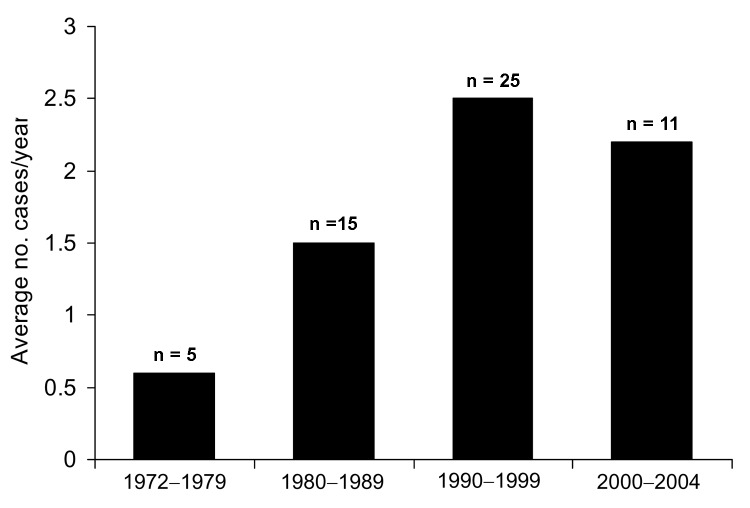
*Capnocytophaga canimorsus* cases (1972–2004); numbers above bars indicate total human cases during the indicated period.

The characteristics of these 56 patients are listed in the Table. The average age was 57.5 years (range 4 months to 99 years); 70% of patients were >50 years of age. Male patients represented 57% of cases. All strains were recovered from adult patients except for 2 blood isolates recovered from 2 infants. One of these infant cases was previously described (*6*). More than 60% of patients from whom *C. canimorsus* was recovered initially had sepsis, a combination of septicemia and meningitis, or a fever of unknown origin. The most commonly reported symptoms associated with these conditions were fever (85%), diarrhea or abdominal pain (21%), vomiting (18%), headache (18%), confusion (12%), and myalgia or malaise (<10%). Disseminated intravascular coagulation (DIC) or septic shock developed in 7 patients (13%) during hospitalization. For 6 patients (11%), the admitting diagnosis was cellulitis; in each instance, *C. canimorsus* was recovered from the blood but not from wounds. In 55 (98%) of 56 cases, *C. canimorsus* was believed to have caused the clinical syndrome. The singular exception was a 19-year-old woman with acute pharyngitis; both *C. canimorsus* and group A streptococcus were isolated from her oropharynx.

Among patients with known risk factors associated with *C. canimorsus* infection, 3 patients were asplenic; no patients with a history of alcohol abuse were identified, although complete medical information was not always available. Other co-existing conditions in these patients included chronic obstructive pulmonary disease, diabetes mellitus, cirrhosis, Grave's disease, hemosiderosis, Hodgkin lymphoma, and ovarian cancer. For 27 patients, records on animal exposure were available. In 21 (78%), a recent history of a dog bite or close contact with dogs or cats was noted (Table). The median time from a dog bite to onset of symptoms was 3 days (range 1–10 days).

**Table:** Clinical data on persons infected with *Capnocytophaga canimorsus*

Characteristic	No. positive (%)
Admitting diagnosis (N = 56)	
Sepsis	18 (32)
Fever of unknown origin	7 (13)
Meningitis	7(13)
Cellulitis	6 (11)
Septic shock	5 (9)
Respiratory tract infections	4 (7)
Phlebitis	1 (2)
Endocarditis	1 (2)
Urosepsis	1 (2)
Septic knee	1 (2)
Diverticulitis	1 (2)
Meningococcemia	1 (2)
Unknown	3 (5)
Sources of isolates (N = 56)	
Blood	49 (88)
Cerebrospinal fluid	4 (7)
Blood and cerebrospinal fluid	2 (4)
Respiratory tract	1 (2)
Animal exposure (n = 27)*	
Dog bite	17 (63)
Close animal contact	3 (11)
Cat scratch	1 (4)
No known exposure	6 (22)
Outcome (n = 30)*	
Survived	20 (67)
Died	10 (33)

*Number of cases for which medical information was available.

Complete or partial medical records were available for 30 cases in which the outcome of infection was recorded. The case-fatality ratio was 33%. Five of the 6 patients with culture-confirmed meningitis survived. Patient 6, a 56-year-old truck driver with meningitis, overwhelming sepsis, and DIC, died, as did all other persons with *C. canimorsus*–associated DIC.

Only one third (32%) of all isolates forwarded to MDL were submitted with the correct species identification. Many strains were received as either an unidentified gram-negative rod or "identification unknown" (55%). In some instances (≈13%), strains were submitted with incorrect identifications, such as *Streptobacillus* spp., anaerobes, *Legionella* spp., or *Haemophilus* spp. Microbiologists continue to have difficulty correctly identifying this organism. From 1998 to the present, only 5 (28%) of 18 *C. canimorsus* strains were correctly identified to genus and species, a slightly lower percentage from that observed for the entire study period.

During the 32-year period, the techniques and methods used by MDL to identify *C. canimorsus* evolved. Recently, 2 cultures received by MDL were nonviable when isolation techniques were attempted from submitted blood culture bottles (1 submitted as a gram-negative rod, the other as *Streptobacillus* sp.). In both instances, however, *C. canimorsus* was identified as the etiologic agent by polymerase chain reaction (PCR) amplification and 16S rRNA gene sequencing. This sequence-based approach is proving increasingly useful for identifying slow-growing, fastidious bacteria, and it can readily differentiate *C. canimorsus* and the phenotypically similar *C. cynodegmi* (*7*).

## Conclusions

Our report describes the single largest series of *C. canimorsus* isolates (N = 56) reported in the medical literature and includes cases of infection from before the species was described (*8*) and before the first case report by Bobo and Newton was published (*9*). *C. canimorsus* isolates have been forwarded to MDL with increasing frequency since 1990 (Figure). The increased frequency associated with *C. canimorsus* may be related to several underlying factors, including more pet (dog, cat) owners, greater opportunities for animal bites (*1**,**2*), and enhanced laboratory techniques to recover this agent from clinical material. However, the accurate identification of this life-threatening pathogen continues to be elusive. The ability of commercial bacterial identification systems to accurately identify these organisms is largely unstudied. Clinical laboratories should consider *C. canimorsus* in patients with bacterial sepsis and a recent history of a dog bite or animal exposure and with the laboratory observation of fastidious, oxidase- and catalase-positive, gram-negative rods with fusiform shape.

The clinical characteristics and demographics of 55 *C. canimorsus*–infected persons closely resembled those described in several other studies or reviews (*3**,**4**,**10*). Most patients were men >50 years of age and had either recently been bitten by a dog or had prolonged contact with dogs. Septic shock and DIC carried a poor prognosis. The observed case-fatality ratio (33%) was comparable to that (30%–31%) found in 2 other surveys (*3**,**4*). Five (83%) of 6 patients with laboratory-confirmed meningitis survived their systemic infections. LeMoal et al. (*11*) recently summarized the literature on case reports of *C. canimorsus* meningitis and found a low death ratio (5%) associated with 19 central nervous system infections; our report supports those observations.

A known risk factor for disseminated *C. canimorsus* infection is asplenia, although this condition could only be demonstrated in 3 (10%) of 31 patients for whom partial or complete medical histories were available. No cases of alcoholism were identified in this series, although several previous series have identified 18%–24% of infected patients with alcoholism as a predisposing factor (*3**,**4*). However, a limitation of the current study was our inability to obtain medical histories on a sizeable number of patients, despite repeated attempts. Lack of such information may considerably bias the data presented.

*C. canimorsus* is a fastidious organism, often difficult to isolate and identify. Identification of isolates may require an extended incubation period (days), delaying laboratory reports and indirectly affecting therapy options and treatment. Many laboratories were unable to presumptively identify *C. canimorsus* isolates, commonly reporting these strains as either gram-negative rods or fastidious gram-negative bacilli. Reasons for mislabeling may include lack of familiarity with the organism, lack of appropriate biochemical tests, or use of commercial identification systems not designed for identifying fastidious microorganisms. These facts, coupled with the low correct identification rate (32%) provided by laboratories in 3 decades of study, suggest that the frequency of *C. canimorsus* infections in the general population may be underestimated, especially if all such generically identified isolates are not forwarded to reference or public health laboratories for definitive identification. Our most recent 2 cases in this study were eventually identified by 16S rRNA gene sequencing and were inadequately or incorrectly identified as other microorganisms by the original submitting laboratories. These cases would have been missed without a molecular approach. Since the case-fatality ratio associated with this infection has remained unchanged, new approaches need to be developed to provide a more rapid and specific diagnosis of this zoonotic pathogen. Such approaches could include 16S rRNA gene sequencing or PCR assays targeting species-specific genes.
